# Aerobic Exercise Improves Methamphetamine-Induced Olfactory Dysfunction Through α-Synuclein Intervention in Male Mice

**DOI:** 10.3389/fnmol.2022.884790

**Published:** 2022-05-02

**Authors:** Zhuo Wang, Rui Zheng, Xiaohan Wang, Xuekun Huang, Jian Huang, Cihang Gu, Yitong He, Shuo Wu, Jingyuan Chen, Qintai Yang, Pingming Qiu

**Affiliations:** ^1^Department of Infertility and Sexual Medicine, The Third Affiliated Hospital of Sun Yat-sen University, Guangzhou, China; ^2^Department of Otorhinolaryngology-Head and Neck Surgery, Department of Allergy, The Third Affiliated Hospital of Sun Yat-sen University, Guangzhou, China; ^3^Guangzhou Key Laboratory of Forensic Multi-Omics for Precision Identification, School of Forensic Medicine, Southern Medical University, Guangzhou, China

**Keywords:** methamphetamine, α-synuclein, exercise, olfactory, mitral cells

## Abstract

Methamphetamine (Meth) is a predominantly abused neurostimulant, and its abuse is often associated with multiple neurological symptoms. Olfaction, the sense of smell, is a highly neurotransmission-dependent physiological process; however, the effect of Meth on olfactory function and its underlying mechanisms remain largely unknown. This study aimed to explore the impact of Meth abuse on the olfactory system and the potential mechanisms. Chronic Meth abuse was induced by daily administration of Meth in male mice for 4 weeks, and we then systematically examined olfactory performance. Behavioral tests found that Meth-treated animals showed increased olfactory threshold, decreased olfactory sensitivity, reduced olfactory-dependent discrimination, and difficulty in seeking buried food. Notably, the increased deposition of α-synuclein (α-syn) in the olfactory bulb was detected. Adeno-associated virus (AAV)-mediated α-syn intervention therapy in the olfactory bulb significantly alleviated Meth-induced olfactory function impairment, and 8 weeks of aerobic exercise showed similar effects through the same principle of α-syn intervention. Notably, exercise-mediated reduction of α-syn inhibited abnormal firing activity and restored the inhibitory synaptic regulation of mitral cells in the olfactory bulb. These findings suggest the involvement of α-syn in the pathogenic mechanisms of Meth-induced olfactory dysfunction and shed light on the possible therapeutic applications of aerobic exercise in Meth-induced olfactory dysfunction.

## Introduction

Methamphetamine (Meth) is a commonly abused psychoactive stimulant, and the related symptoms have been widely recognized, with a heavy social burden (Courtney and Ray, 2014). Chronic Meth administration leads to adverse effects on various types of neural cells across different brain regions and induces neuropsychiatric symptoms, such as addiction, emotional disorders, cognitive impairment, and Parkinson’s disease (PD) (Cruickshank and Dyer, 2009; Büttner, 2011). However, the effects of Meth on basic sensory systems, especially olfaction, are still less known and often overlooked.

Among the sensory systems, olfaction is one of the most evolutionarily conserved neural-processing senses and is crucial for survival across species. Olfaction is involved in multiple aspects of social behaviors, such as emotion control, reward activation, territory defense, social recognition, and mate selection (Kaupp, 2010; Pinto, 2011; Kavaliers et al., 2020). Notably, olfactory dysfunction is very common among populations with substance abuse, and more than 52% of substance abusers have reported abnormalities in olfactory performance (Podskarbi-Fayette et al., 2005). In Meth abusers, abnormalities of olfactory function, such as olfactory hallucinations, are common psychiatric complaints (Mahoney et al., 2010; McKetin et al., 2017). In animal studies, chronic Meth exposure has been found to induce alterations in the expression of proteins involved in processes such as neuroinflammation and neurodisability in the olfactory bulb (Zhu et al., 2016). In addition, acute Meth administration results in impaired socially/non-socially dependent olfactory discrimination in rodents (O’Dell et al., 2011; Ramkissoon and Wells, 2015). However, these studies could not distinguish whether the reduced olfactory recognition performance is caused by the impairment of olfactory cognitive function or olfaction alone, nor could they reveal the olfactory function and its underlying mechanism in drug abusers in chronic conditions.

Alpha-synuclein (α-syn, encoded by *SNCA*) is a soluble cytosolic neuronal protein belonging to the synuclein family (Sulzer and Edwards, 2019). Accumulating studies have revealed that α-syn is involved in maintaining normal synaptic transmission and is implicated in the pathogenesis of synucleinopathies (Burré et al., 2018), and the olfactory bulb is not only the brain region with high expression of α-syn (Hansen et al., 2013) but also the initial site of its propagation to multiple brain regions (Cersosimo, 2018). The accumulation of α-syn in the olfactory bulb is increased during aging and in neurodegenerative diseases (Bobela et al., 2015; Srinivasan et al., 2021), and artificial regulation of systemic/brain α-syn expression or direct overexpression of α-syn in the olfactory bulb has been shown to cause olfactory impairment (Hansen et al., 2013; Zhang et al., 2015; Chen et al., 2021). Notably, a definite association has been found between abnormal metabolism of α-syn and the occurrence of neuropsychiatric symptoms (Wu et al., 2021). However, whether and how Meth affects α-syn in the olfactory bulb remain unknown. According to anatomical and immunohistochemical results, α-syn is mainly distributed in the mitral/granulosa cell layer of the olfactory bulb (Hansen et al., 2013). In the olfactory system of information processing, the smell signal first activates olfactory sensory neurons in the nasal epithelium, and the activated olfactory sensory neurons can stimulate the olfactory bulb mitral cells. After processing and encoding olfactory information, mitral cells project olfactory information through axons to the higher olfactory cortex (Nunez-Parra et al., 2014). The fine regulation of mitral and granule cells in the olfactory bulb and their normal electrophysiological performance play an important role in the correct processing and transmission of olfactory information. However, when α-syn accumulates in the olfactory bulb, local olfactory bulb neural activity is perturbed (Kulkarni et al., 2020). These results imply the effect of Meth on olfactory function and its potential α-syn-involved mechanism.

Aerobic exercise, which has been widely used as rehabilitation therapy, has shown a protective role in brain functional recovery among Meth-dependent abusers (Zhang et al., 2018; Huang et al., 2020; Liu et al., 2021). In rodents, voluntary wheel running mimics long-term regular aerobic exercise with adequate oxygen supply (Manzanares et al., 2018). More intriguingly, aerobic exercise has been found to promote α-syn clearance and improve recovery of olfactory function (Koo and Cho, 2017; Tian et al., 2020). This finding has prompted our interest in exploring the effect of aerobic exercise on the potential impairment of olfactory function caused by chronic Meth administration. Accordingly, in this study, we investigated the effects of chronic Meth abuse on olfactory function and explored the potential mechanisms by which aerobic exercise improves Meth abuse-induced olfactory dysfunction.

## Materials and Methods

### Animals

Male c57BL/6 mice aged 8–10 weeks and weighing 20–25 g were used in this study. The experimental animals were supplied by the Laboratory Animal Center of Southern Medical University (Guangzhou, Guangdong, China). Animals were raised in a standard specific pathogen-free experimental environment with free access to water and food, constant temperature (23 ± 1°C) and humidity (50–60%), and dark and light for 12 h each. All experimental procedures were carried out in accordance with the Principles of Laboratory Animal Care (NIH Publication no. 85–23, revised 1985) and supervised by the Animal Ethics Committee of The Third Affiliated Hospital of Sun Yat-sen University. The chronic administration route and dosage (10 mg/kg i. p. daily, National Institute Control of Pharmaceutical and Biological Products, Beijing, China) of Meth were chosen based on our previous chronic toxicity study (Wang et al., 2021a). Behavioral experiments were performed 1 week after the last Meth administration or 4 weeks after the virus injection. The exercise method utilized in this study was voluntary wheel-running and adapted from published procedures (Jang et al., 2017; El Hayek et al., 2019). Briefly, mice used for exercise training were housed individually and allowed free access to a steel running (rotatable/locked) wheel for 8 weeks. Upon completion of the behavioral experiments, the mice were deeply anesthetized with sodium pentobarbital for sample collection and electrophysiological recordings. Every possible effort was made to minimize animal pain or discomfort and reduce the number of animals used.

### Behavioral Test

The assessment of mouse olfactory behavior was performed with a digital video recording device in the zenithal position by an experienced double-blind researcher. Different experimental paradigms depict different perspectives on olfactory performance.

**FIGURE 1:**
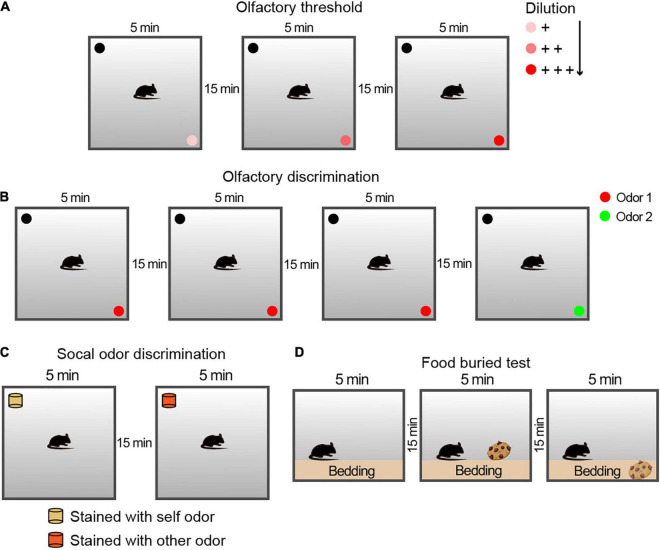
Schematic diagram of the protocol of the behavioral experiment for the olfactory examination. **(A)** Experimental configuration for the olfactory threshold and sensitivity tests. Each trial lasted for 5 min, followed by a 15-min rest interval. For the avoidance and preference tests, three solutions of different concentrations were used. **(B)** Experimental configuration for the olfactory discrimination test. Each trial lasted 5 min, followed by a 15-min rest interval. For the olfactory discrimination test, two different odor solutions were used. **(C)** Schematic drawing of the experimental steps for the social olfactory discrimination test. Each trial lasted 5 min, followed by a 15-min rest interval. The blocks were placed consistently above the bedding between cages in the corresponding home cages. For social odor discrimination, mice were given a comparison between blocks with their own odor and those with the odor of an unfamiliar conspecific male. **(D)** Schematic drawing of the experimental steps for the buried food test for olfactory function examination. Each trial lasted 3 min, followed by a 15-min rest interval. The cookies were placed above or buried below the bedding in the corresponding two stages.

The olfactory threshold examination, which uses rodents’ innate conditioned preference/aversion responses to odors, was performed and adapted as previously described by Breton-Provencher et al. (2009). The olfactory threshold and sensitivity of animals can be measured by observing their responses to different concentrations of odors. In this study, butyraldehyde was used as an aversive odor, and limonene was used as a preferred odor. During a 5 min period, each animal was allowed to explore different odorant solutions at different concentrations in three independent experiments with odorless solvent as a control, and the preference index was calculated (see Figure 1A for schematic diagram).

The olfactory discrimination habituation/dishabituation test was performed as described previously (Gheusi et al., 2000) to assess whether the animal could differentiate between different odors. Each session consisted of three repetitions of exploration with the same odor (Odor 1) as the familiar condition and a new odor (Odor 2) as a novel condition (see Figure 1B for schematic diagram).

Social odor discrimination was used to identify whether animals can discriminate between different social odors (Petit et al., 2013). The odorless blocks were placed for 24 h in cages with different animals (came from the tested animal or the conspecific stranger of the same sex). The time spent by the mouse sniffing exploration during a 5 min section with an 15 min interval of the block scented of its own and an exploration of novel social scent were matched and measured.

The non-social food buried test was adapted from a previous study (Wu et al., 2016). Mice were fasted for 48 h to ensure sufficient motivation to search for food. The experiment was divided into three 5-min stages, with an interval of 15 min. In the first stage, the animals were placed on the bedding and acclimatized to the environment. In the second stage (visible), a cookie was placed on the bedding of one corner, and the time the mice used to find the food and nibble it within 5 min was recorded. In the third stage (invisible), the food was buried 1.5 cm below the bedding in a randomly selected corner, and the time the animal used to find and nibble it was recorded.

### Immunofluorescence

Immunohistochemistry assays were performed as previously described (Wang et al., 2021b), and mice were transcardially perfused with pre-cooled phosphate-buffered saline (PBS) and 4% paraformaldehyde. The brains were further fixed for 12 h at 4°C and dehydrated in 30% sucrose for cryoprotection. They were then cut into 20-mm-thick sections, and the sections were permeabilized with 0.3% Triton X-100 for 10 min and blocked with 5% bovine serum albumin (BSA) in PBS for 2 h. These brain sections were incubated with primary antibodies against α-syn (SC-7011, 1:1500, Santa Cruz Biotechnology, CA, United States) overnight at 4°C in PBS with 2% BSA. Thereafter, the brain sections were incubated with a secondary Alexa Fluor^®^ 488-conjugated antibody (Invitrogen) for 1 h in the dark. After washing with PBS, 4′,6-diamidino-2-phenylindole (Vector Lab) was used to stain the nuclei, and images were then acquired using a Leica fluorescence microscope.

### Western Blotting

Western blotting was performed after the behavioral experiment, and behavioral and expression data were analyzed for each animal individually. Total proteins were extracted from the olfactory bulb tissues using RIPA lysis buffer (Solarbio, Beijing, China), and the protein concentrations were measured using a Pierce™ BCA protein kit (23227, Thermo Fisher Scientific, Waltham, MA, United States). Equal amounts of protein lysates (approximately 40 μg) were separated using 12% sodium dodecyl sulfate polyacrylamide gel electrophoresis and subsequently transferred to polyvinylidene difluoride membranes (Millipore, Bedford, MA, United States). The membranes were incubated with 5% BSA to block non-specific binding. Next, the membranes were probed with specific primary monoclonal antibodies at 4°C overnight, including anti-α-syn (SC-7011, 1:3000, Santa Cruz Biotechnology) and glyceraldehyde 3-phosphate dehydrogenase (GAPDH, ab9485, 1:3000, Abcam). Subsequently, the membranes were incubated with HRP-labeled anti-rabbit IgG (ab288151, Abcam) at 37°C for 2 h. Finally, protein expression was visualized using enhanced chemiluminescence reagents (Thermo Fisher Scientific). GAPDH was used as an internal reference for normalization.

### Viral Injection

Stereotaxic surgeries were performed as described previously (Wang et al., 2019). Under isoflurane gas anesthesia, mice were placed in a stereotaxic frame, and the skull was exposed and adjusted to ensure that the bregma and the lambda axis were horizontal. For α-syn (*Snca*) knockdown and overexpression, 1 μL of EGFP-tagged rAAV-α-syn (AAV-α-syn, 3.4E_12 V. g/L), rAAV-control (AAV-Con, 4.2E_12 V.G./ml), rAAV-α-synuclein-shRNA (α-syn-shRNA, 6.3E_12 V. g/mL), or rAAV-control-shRNA (Con-shRNA, 6.2E_12 V.G./ml) was bilaterally injected into the olfactory bulb (coordinates from bregma AP: 4.5 mm; ML: ± 0.75 mm; DV: −3.25 mm), and the needle was withdrawn gently 10 min after injection. Viral vectors were designed and constructed using a commercial packaging service (Brain Case Co., Ltd., Shenzhen, China). Mice with missed injections or EGFP expression outside the olfactory bulb were excluded from the analysis after *post-hoc* examination.

### Electrophysiological Recording

The animals were euthanized by carbon dioxide asphyxiation and decapitated, and their brains were rapidly and gently removed. The olfactory bulb was then sliced into 300-μm-thick sections with a vibrating tissue slicer (World Precision Instruments Vibroslicer, Sarasota, FL, United States), and the sections were placed in a pre-cooled artificial cerebrospinal fluid (ACSF) circulating with 95% O_2_ and 5% CO_2_. The ACSF solution contains 124 mmol/L NaCl, 24 mmol/L NaHCO_3_, 5 mmol/L KCl, 2.4 mmol/L CaCl_2_, 1.3 mmol/L MgSO_4_, 1.2 mmol/L KH2PO_4_, and 10 mmol/L glucose (pH = 7. 4). Recordings began after at least 1 h of recovery. During recording, the olfactory bulb sections were transferred from the incubation tank to the recording tank and fixed with a brain slice fixator under light pressure. The recording tank was continuously filled with 95%O_2_/5%CO_2_-ventilated ACSF, which was prepared with ACSF in advance when certain drugs were applied, and the ACSF was replaced through the fluid inlet of the perfusion system. The irrigation rate was 2 mL/min, and the temperature of the recording tank was maintained at 29–30°C with a heating rod. Mitral cells in the olfactory bulb were identified by position, morphology, and electrical characteristics under a 60 × water-immersion objective microscope (Schoppa, 2006). For whole-cell action potential recording, pipettes were filled with a solution containing 130 mmol/L K-Gluconate, 20 mmol/L KCl, 10 mmol/L HEPES, 4 mmol/L MgATP, 10 mmol/L Na-Phosphocreatine, 0.3 mmol/L Na_2_GTP, and 0.5 mmol/L EGTA, adjusted to pH 7.4 with KOH. The miniature inhibitory postsynaptic currents (mIPSCs) were monitored in the presence of tetrodotoxin to block action potentials. For mIPSC recordings, the intracellular solution contained 125 mmol/L KCl, 3 mmol/L Mg-ATP, 1 mmol/L MgCl_2_, 10 mmol/L HEPES, 0.02 mmol/L EGTA, and 0.5 mmol/L Na_3_-GTP. All data acquisition and analysis were performed using a Multiclamp 700 b amplifier (Cellular Devices, Sunnyvale, CA, United States), pClamp 10 (Molecular Devices, Sunnyvale, CA, United States), and Mini Analysis (Synaptosoft, Fort Lee, NJ, United States).

### Statistical Analysis

GraphPad Prism 6.00 was used for data processing. All experimental results are expressed as mean ± SEM. Student’s *t*-test was used to analyze the difference between two groups, one-way analysis of variance (ANOVA) among ≥three groups, and two-way ANOVA between factorial designed groups. A linear relation was analyzed by Spearman’s correlation coefficient. The sample size in each group is indicated in each figure legend. *P* < 0.05 represented a significant difference.

## Results

### Chronic Methamphetamine Administration Induces Severe Olfactory Functional Deficits in Mice

**FIGURE 2:**
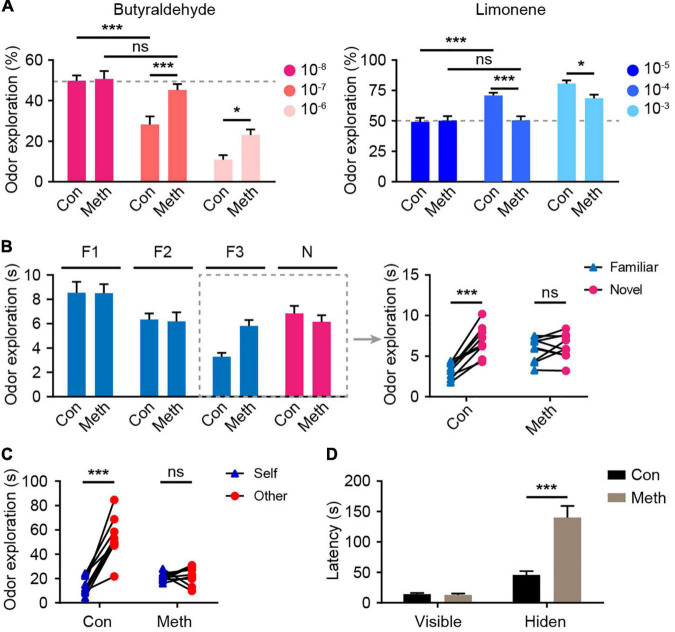
Chronic methamphetamine administration induces impairment of olfactory performance. **(A)** Chronic Meth administration decreased olfactory sensitivity to both aversive and favorite odors. Left, olfactory avoidance performance for an aversive odor (butyraldehyde). The concentrations of the butyraldehyde solution ranged from 10^– 8^ to 10^– 6^ (*n* = 9). *F*_(2, 32)_ = 4.246, *P* = 0.0231 by two-way RM ANOVA with Bonferroni’s multiple comparisons test. Right, olfactory sensitivity to a favorite odor (Limonene). The concentrations of limonene solution ranged from 10^– 5^ to 10^– 3^ (*n* = 9). *F*_(2, 32)_ = 5.601, *P* = 0.0082 by two-way RM ANOVA with Bonferroni’s multiple comparisons test. **(B)** Chronic Meth administration impaired olfactory discrimination. Left, statistical analysis for the experiments that were performed independently four times. Right, the corresponding exploration time of each animal for different odors in the dashed rectangular line (*n* = 9). *F*_(1, 16)_ = 23.49, *P* = 0.0002 by two-way RM ANOVA. **(C)** Chronic Meth administration impaired social olfactory discrimination. Scatter dots represent animals, and they were paired with the corresponding strangers (*n* = 9). *F*_(1, 16)_ = 37.13, *P* < 0.0001 by two-way RM ANOVA. **(D)** Chronic Meth administration impaired olfactory function in locating food (*n* = 9). *F*_(1, 16)_ = 23.09, *P* = 0.0002 by two-way RM ANOVA. F, familiar; N, novel; ns, not significant. Data are presented as mean ± SEM. *P < 0.05; ***P < 0.001.

To evaluate the effect of Meth on olfactory function, the overall olfactory performance was estimated using a series of statistical indicators, including olfactory threshold, olfactory discrimination, social odor discrimination, and food exploration. The behavioral research strategy is illustrated in Figure 1. Behavioral tests were conducted after 4 weeks of administration of Meth. Generally, animals display an inborn aversion or favorite to the conditioned odor. Thus, animals were tested for their avoidance or preference for a certain solution. Butyraldehyde and limonene were gradient-diluted to test the sensitivity of the animal to avoidance or preference. We found that chronic Meth administration significantly decreased olfactory sensitivity in both the avoidance and preference tests (Figure 2A); higher odor concentration was required to show preference/aversion, and the preference/aversion degree was lessened under the same odor concentration. In another olfactory test, which involved olfactory cognitive function, the animals were exposed to the same odor three times before being exposed to a novel odor in the fourth test (Figure 2B). With familiarity with the same smell, the mice in the control group showed a decrease in the desire to explore the familiar odor, while a significant increase was observed after giving new odors. This process was impaired in animals administered with chronic Meth. We also examined the social-related olfactory function (Figure 2C), and the control animals showed significant interest in exploring blocks that had been contaminated with the scent of a congeneric stranger, while animals with chronic Meth administration showed obvious defects in this test. Finally, we assessed the ability of olfactory-dependent food acquisition (Figure 2D). When cookies were placed on the bedding, the two groups of animals could see food and showed a similar latency to eat. When the food was buried under the bedding, animals with chronic Meth administration took a longer time to find the food. These results indicate that chronic Meth administration induces olfactory dysfunction.

### Chronic Methamphetamine Administration Increases the Expression of α-Synuclein in the Olfactory Bulb in Mice

**FIGURE 3:**
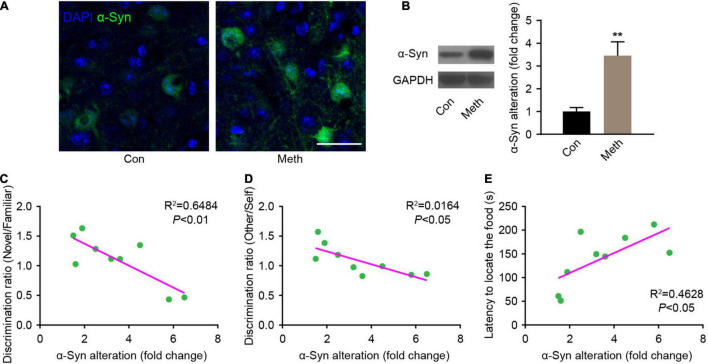
Chronic methamphetamine administration increases the expression of α-syn in the olfactory bulb. **(A)** Representative confocal immunofluorescence images of the OB mitral cells stained for α-syn (Green) upon chronic methamphetamine administration. Scale bar, 20 μm. **(B)** The levels of α-syn were examined by western blotting (*n* = 9). *t* = 3.895 by unpaired t test. GAPDH served as a normalization control. The correlation between α-syn expression changes and olfactory discrimination **(C)**, social olfactory discrimination **(D)** and buried food test **(E)** results was analyzed in chronic methamphetamine-administered mice, and there was a significant association between α-syn changes and behavioral alterations (*n* = 9). Data are presented as mean ± SEM. **P < 0.01.

Methamphetamine can promote the accumulation of α-syn, and the olfactory bulb is the core brain region of α-syn metabolism in the central nervous system (Rey et al., 2013, 2016; Zhu et al., 2018). Therefore, α-syn expression in the olfactory bulb was detected. Immunofluorescence staining showed that the presence of α-syn was remarkably increased after chronic Meth administration (Figure 3A), and western blot analysis confirmed the same effect (Figure 3B). Correlation analysis of the behavioral and western blotting results showed that the increase in α-syn was negatively correlated with the non-socially/socially odor discrimination ratio (Figures 3C,D), and positively correlated with the degree of olfactory function impairment (Figure 3E). This finding suggests a causal relationship between the increased expression of α-syn and the appearance of olfactory impairment.

### Inhibition of α-Synuclein Prevents Methamphetamine-Induced Olfactory Deficits

**FIGURE 4:**
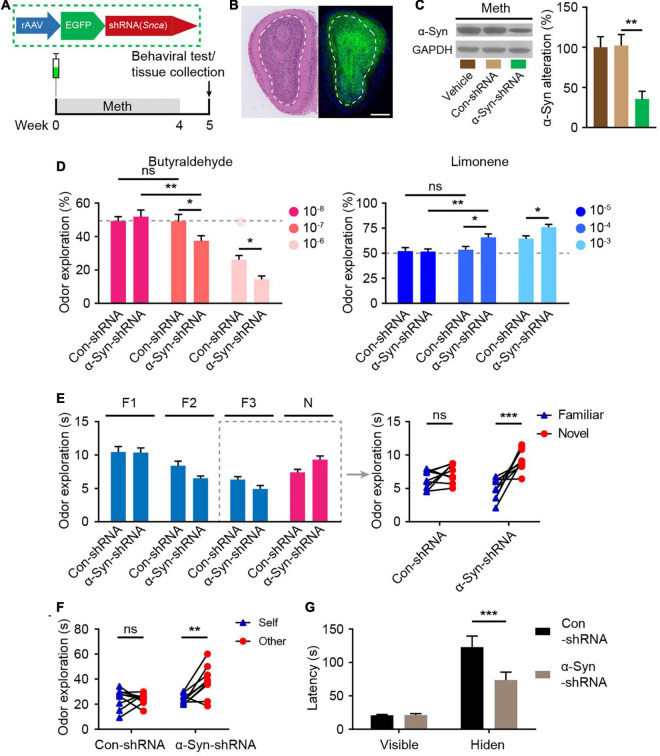
Suppression of α-syn rescues methamphetamine-induced olfactory behavioral abnormalities. **(A)** Experimental scheme for injection of reconstructed adeno-associated virus (rAAV) containing α-syn-shRNA or control into the olfactory bulb and subsequent behavioral testing. **(B)** Schematic representation of the rAAV injection strategy and representative HE staining and immunofluorescence images of olfactory bulb sections of rAAV-injected mice (*n* = 3). Scale bar, 500 μm. **(C)** The efficiency of α-syn-shRNA was determined by western blot analysis (*n* = 6). *F* = 9.371, *P* = 0.0023 by One-way ANOVA with Bonferroni’s multiple comparisons test. **(D)** Suppression of α-syn increased olfactory sensitivity to both aversive (butyraldehyde) and favorite (Limonene) odors (*n* = 9). Butyraldehyde: *F*_(2, 32)_ = 3.383, *P* = 0.0465 by two-way RM ANOVA with Bonferroni’s multiple comparisons test. Limonene: *F*_(2, 32)_ = 2.926, *P* = 0.0681 by two-way RM ANOVA with Bonferroni’s multiple comparisons test. **(E)** Suppression of α-syn improved olfactory discrimination (*n* = 9). *F*_(1, 16)_ = 8.432, *P* = 0.0104 by two-way RM ANOVA. **(F)** Suppression of α-syn improved social olfactory discrimination. Each scatter dot represents an animal, and it was paired with the corresponding stranger (*n* = 9). *F*_(1, 16)_ = 7.993, *P* = 0.0121 by two-way RM ANOVA. **(G)** Suppression of α-syn improved olfactory function to locate invisible food (*n* = 9). *F*_(1, 16)_ = 6.669, *P* = 0.0200 by two-way RM ANOVA. F, familiar; N, novel; ns, not significant. Data are presented as mean ± SEM. *P < 0.05; **P < 0.01; ***P < 0.001.

To investigate the role of α-syn in the regulation of olfactory functional deficits following chronic Meth administration, olfactory bulb neurons were infected with rAAVs expressing shRNA against α-syn (α-syn-shRNA) or non-targeting scrambled sequence shRNA (Con-shRNA) as a control before the administration of Meth (Figure 4A). The injection accuracy and infection efficiency of rAAV were examined by immunofluorescence (Figure 4B), and a significant decrease in α-syn expression levels within olfactory bulb regions was successfully identified in α-syn-knockdown mice (Figure 4C). Knockdown of α-syn within the olfactory bulb significantly ameliorated Meth-induced olfactory deficits, as evidenced by increases in olfactory sensitivity in both the avoidance and preference tests (Figure 4D), novel odor discrimination (Figure 4E), social-related olfactory function (Figure 4F), and olfactory-dependent food acquisition (Figure 4G). These findings demonstrate that reducing the abnormal deposition of α-syn in the olfactory bulb ameliorates chronic Meth administration-induced olfactory dysfunction.

### Exercise Alleviates Methamphetamine-Induced Olfactory Dysfunction Through α-Synuclein Intervention

**FIGURE 5:**
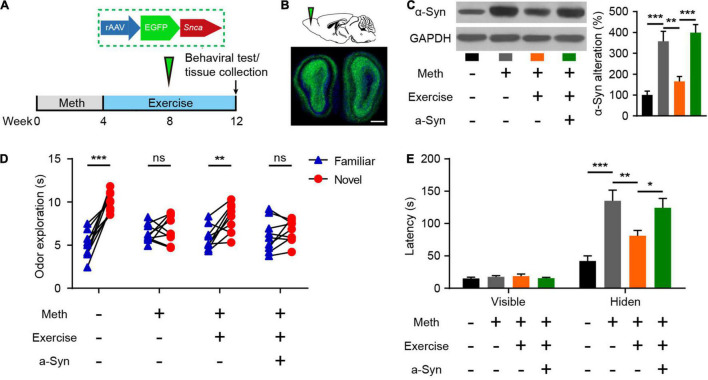
Exercise improves methamphetamine-induced olfactory behavioral abnormalities through reduced α-syn expression. **(A)** Illustration of rAAV packed with human SNCA and experimental paradigm for methamphetamine administration, virus injection, and behavioral testing. **(B)** Schematic representation of rAAV-α-syn injection and GFP fluorescence in the infected region of the olfactory bulb. Scale bar, 500 μm. **(C)** Effects of exercise and olfactory bulb rAAV-α-syn injection on α-syn expression were determined by western blot analysis. *F* = 17.98, *P* < 0.0001 by One-way ANOVA with Bonferroni’s multiple comparisons test. **(D)**: Effects of exercise and olfactory bulb rAAV-α-syn injection on olfactory discrimination (*n* = 9). *F*_(3, 32)_ = 11.47, *P* < 0.0001 by two-way RM ANOVA with Bonferroni’s multiple comparisons test. **(E)** Effects of exercise and olfactory bulb rAAV-α-syn injection on locating invisible food (*n* = 9). *F*_(3, 48)_ = 9.765, *P* < 0.0001 by two-way RM ANOVA with Bonferroni’s multiple comparisons test. Data are presented as mean ± SEM. *P < 0.05; **P < 0.01; ***P < 0.001; ns, non-significant.

Exercise has been recognized to combat the adverse effects of drug use and is closely associated with improved olfactory function as we have mentioned above. Here, we examined the effects of exercise on the olfactory dysfunction induced by chronic Meth. Furthermore, to test whether recovery of α-syn expression could block the effect of exercise, we generated rAAVs containing α-syn or GFP and injected them correspondingly during the exercise (Figures 5A,B). Eight weeks of aerobic exercise not only reduced the amount of α-syn deposited in the olfactory bulbs but also improved olfactory function (Figures 5C–E). However, overexpression of human α-syn blocked the alleviating effect of exercise on Meth-induced olfactory dysfunction (Figures 5C–E).

### Exercise Improves Impaired Inhibitory Synaptic Transmission in the Olfactory Bulb

**FIGURE 6:**
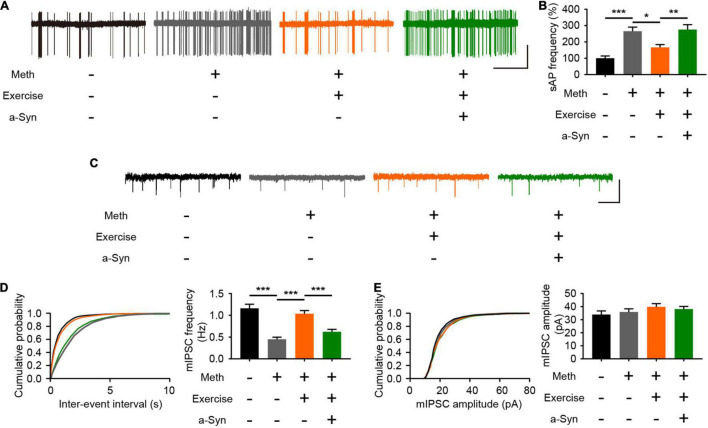
Exercise improves methamphetamine-induced impaired inhibitory synaptic transmission in the olfactory bulb through α-syn intervention. **(A)** Representative traces of spontaneous action potential of mitral cells. Scale bar, 25 pA, 5 s. **(B)** Quantitative analysis of spontaneous firing rates of mitral cells in each group (a total of 12 cells from 3 to 4 mice were recorded in each group). *F* = 13.87, *P* < 0.0001 by One-way ANOVA with Bonferroni’s multiple comparisons test. **(C)** Representative traces of whole-cell voltage-clamp recordings showing mIPSC events from mitral cells in olfactory bulb slices. Scale bar, 100 pA, 1 s. **(D)** Cumulative plots of the mIPSC interevent interval and quantitative analysis of mIPSC frequency in mitral cells (a total of 15 cells from four to five mice were recorded in each group). *F* = 22.41, *P* < 0.0001 by One-way ANOVA with Bonferroni’s multiple comparisons test. **(E)** Cumulative plots of the mIPSC amplitude and quantitative analysis of mIPSC amplitude in mitral cells (a total of 15 cells from four to five mice were recorded in each group). *F* = 1.078, *P* = 0.3659 by One-way ANOVA with Bonferroni’s multiple comparisons test. Data are presented as mean ± SEM. *P < 0.05; **P < 0.01; ***P < 0.001.

Normal neural activity and synaptic plasticity of mitral cells are required for the efficient performance of olfactory function, and the accumulation of α-syn is tightly coupled with abnormal electrical activity of olfactory neurons (Chen et al., 2021). According to the cell-attached recordings, Meth increased the spontaneous firing rate of mitral cells; this effect could be alleviated by exercise but blocked upon α-syn restoration (Figures 6A,B). The spontaneous firing activity of mitral cells is controlled by local inhibitory synaptic drive (Nai et al., 2009). Thus, we measured the level of mIPSCs in mitral neurons. We found that Meth administration caused a significant decrease in the frequency (but not in the amplitude) of mIPSCs (Figures 6C–E). As expected, exercise led to upregulation in the frequency of mIPSCs, and this effect was blocked by rAAV-α-syn injection; no significant changes were observed in the amplitude of mIPSCs (Figures 6C–E). These results suggest that exercise could reverses the impaired inhibitory synaptic transmission in the olfactory bulb through a α-syn-selective manner.

## Discussion

In this study, we systematically examined the effects of Meth on olfactory performance and demonstrated that Meth could impair olfactory function through upregulated α-syn levels in the olfactory bulb. Aerobic exercise could reverse this process by implementing α-syn intervention and restoring inhibitory synaptic transmission of mitral cells. These results deepen our understanding of Meth-induced neurotoxicity and provide new insights into the pathogenesis and treatment of meth-related encephalopathy.

Olfaction is the physiological basis of many biological behaviors (Mori and Sakano, 2021), and its dysfunction is a common symptom that occurs early in many neuropsychiatric diseases (Waldmann et al., 2020). In fact, many neuropsychiatric symptoms induced by Meth, such as aggressive behavior (Stowers et al., 2013), depressed mood (Athanassi et al., 2021), and olfactory hallucination (Mahoney et al., 2010), are all strongly linked to olfaction. Olfactory function is important but often neglected during physical examination in Meth users, and the damage to the nasal mucous membrane after long-term snorting/intranasal route administration may cause olfactory function damage naturally. However, the effects of Meth on the entire olfactory pathway have not been revealed. Thus, we employed intraperitoneal injection delivery methods, which avoid direct adverse stimulation to the nasal mucosa and make it possible to explore the effects of Meth on the central olfactory system. Meth can cause severe cognitive dysfunction (Mizoguchi and Yamada, 2019); therefore, the influence of olfactory-related cognitive factors on olfactory behavior should be excluded when designing olfactory function examinations. Accordingly, we used the animals’ instinctive aversion to malodorous odors and preference for fresh odors to rule out olfactory cognitive function as an intervening factor. We showed that chronic Meth administration significantly reduced the olfactory threshold and sensitivity of animals to averting and favoring smells, and olfactory cognitive function was also impaired, as manifested by impaired social/non-social odor discrimination and a decline in olfactory-dependent food exploration ability. These results suggest olfactory information processing abnormalities in Meth-abused animals.

Previous studies have demonstrated that Meth can induce significant changes in α-syn metabolism in the brain (Mauceli et al., 2006; Wu et al., 2021), and knockdown of α-syn could alleviate pathological brain injury caused by Meth (Ding et al., 2020). However, the behavioral manifestations remain unclear. We hypothesized that α-syn might also mediate the impairment of olfactory bulb function, and our results confirmed this hypothesis. Our findings indicate that the elevation of α-syn is implicated in Meth-induced olfactory dysfunction, and the dysfunction can be alleviated by knockdown of α-syn. Consistently, heterologous injection of α-syn into the olfactory bulb has also been shown to cause abnormalities in olfactory function (Niu et al., 2018; Chen et al., 2021); however, the artificial intervention could not mimic the natural process of disease progression. In this study, we demonstrated for the first time that Meth could impair olfactory function by increasing the levels of α-syn in the olfactory bulb; this is a potential pathophysiological mechanism by which Meth abuse influences olfactory function. An important question that remains to be explored is the exact source of increased α-syn. Meth abuse may not only promote the expression of α-syn in the olfactory bulb, and the increase in peripheral entry and the increased blood-brain barrier permeability may also contribute to the increased olfactory bulb α-syn levels induced by Meth. These hypotheses need to be further verified by subsequent experiments.

Normal synaptic transmission of olfactory bulb neurons plays an important role in olfactory information processing. Our electrophysiological results revealed that mitral cells with impaired inhibitory synaptic transmission exhibited higher cluster discharge activity. The increased firing activity of mitral cells induced by Meth is consistent with the result of direct injection of α-syn into the olfactory bulb (Chen et al., 2021), and similar damage to the inhibitory regulation of mitral cells has also been found in other neurodegenerative diseases, such as Alzheimer’s disease (AD) (Hu et al., 2021). Parallel to the results, the inhibitory synaptic transmission manifested by inhibitory postsynaptic currents (IPSCs) was impaired, thus impairing local inhibitory circuits. α-syn is soluble and can be transported across brain regions in an axon-dependent manner (Li et al., 2004; Kuznetsov and Kuznetsov, 2016). Previous studies have confirmed the existence of α-syn transmission initiated from the olfactory bulb (Cersosimo, 2018). Mitral cells have axons, whereas granule cells are GABAergic interneurons without long projective axons. The spatiotemporal transfer of α-syn in mitral cells can be realized through axons, whereas in granulosa cells, α-syn cannot be translocated across brain regions, probably resulting in cytotoxicity, which may account for the damage of inhibitory synaptic transmissions. Moreover, increased mitral cell neuronal firing activity leads to more and faster transport of α-syn deposited in the olfactory bulb to other brain regions, increasing the risk of neural damage in downstream brain regions. This speculation is consistent with the clinical data and could explain, at least partially, the observed higher risk of early onset Meth-induced neurodegenerative diseases, such as PD (Lappin et al., 2018; Lappin and Darke, 2021). Whether α-syn intervention in the olfactory bulb can delay the development of subsequent neurodegeneration and alleviate related symptoms deserves further exploration.

Exercise is a natural intervention that has been widely implemented in recent years and has been proven to be effective in improving Meth-related neuropsychiatric symptoms. Based on the pathologically related mechanisms of α-syn revealed above, exercise strategies can refer to PD-related intervention training approaches. Indeed, both epidemiological and basic studies have found that aerobic exercise is an effective intervention for synucleinopathies, such as PD (Liu et al., 2020). Therefore, we chose running wheels, which is a widely accepted type of aerobic training. By locking the running wheel, the results of the experiment could eliminate the interference factors of the enriched environment to create a good contrast. This natural alternative therapy reduces the potential risks associated with prescription drugs. Notably, in this study, we revealed the protective effect of exercise on Meth-induced olfactory function impairment through α-synuclein intervention. The findings deepen the understanding of exercise against Meth-induced neurotoxicity.

## Conclusion

In conclusion, our results indicate that Meth can impair olfactory function through the accumulation of α-syn in the olfactory bulb, leading to a state of “olfactory dementia.” Moreover, the aggregation of α-syn induced by Meth affects the inhibitory synaptic input of mitral cells in the olfactory bulb, and exercise could improve olfactory function by rescuing these pathophysiological dysfunctions. The findings provide further evidence of olfactory abnormalities in the development of neurodegenerative diseases and confirm that α-syn in the olfactory bulb is an effective biomarker and therapeutic target for Meth-induced olfactory dysfunction. This study confirms the potential applications of aerobic exercise strategies in substance abusers.

## Data Availability Statement

The raw data supporting the conclusions of this article will be made available by the authors, without undue reservation.

## Ethics Statement

The animal study was reviewed and approved by the Animal Ethics Committee of The Third Affiliated Hospital of Sun Yat-sen University.

## Author Contributions

PQ and QY designed and supervised the study. ZW, RZ, XW, XH, JH, CG, YH, SW, and JC collected, analyzed, and interpreted the data. ZW and RZ drafted the manuscript. PQ, QY, and XH revised the manuscript. All co-authors revised and approved the submitted version.

## Conflict of Interest

The authors declare that the research was conducted in the absence of any commercial or financial relationships that could be construed as a potential conflict of interest.

## Publisher’s Note

All claims expressed in this article are solely those of the authors and do not necessarily represent those of their affiliated organizations, or those of the publisher, the editors and the reviewers. Any product that may be evaluated in this article, or claim that may be made by its manufacturer, is not guaranteed or endorsed by the publisher.
